# Estimating influenza attack rates in the United States using a participatory cohort

**DOI:** 10.1038/srep09540

**Published:** 2015-04-02

**Authors:** Rumi Chunara, Edward Goldstein, Oscar Patterson-Lomba, John S. Brownstein

**Affiliations:** 1 The Global Institute of Public Health, New york University and Computer science & Engineering, New york University; 2 Center for Communicable Disease Dynamics, Department of Epidemiology, Harvard School of Public Health, Boston, Massachusetts, United States of America; 3 Department of Biostatistics, Harvard School of Public Health, Boston, Massachusetts, United States of America; 4 Department of Pediatrics, Harvard Medical School, Boston, Massachusetts, United States of America; 5 Informatics Program, Division of Emergency Medicine, Department of Medicine, Boston Children's Hospital, Boston, Massachusetts, United States of America

## Abstract

We considered how participatory syndromic surveillance data can be used to estimate influenza attack rates during the 2012–2013 and 2013–2014 seasons in the United States. Our inference is based on assessing the difference in the rates of self-reported influenza-like illness (ILI, defined as presence of fever and cough/sore throat) among the survey participants during periods of active vs. low influenza circulation as well as estimating the probability of self-reported ILI for influenza cases. Here, we combined Flu Near You data with additional sources (Hong Kong household studies of symptoms of influenza cases and the U.S. Centers for Disease Control and Prevention estimates of vaccine coverage and effectiveness) to estimate influenza attack rates. The estimated influenza attack rate for the early vaccinated Flu Near You members (vaccination reported by week 45) aged 20–64 between calendar weeks 47–12 was 14.7%(95% CI(5.9%,24.1%)) for the 2012–2013 season and 3.6%(−3.3%,10.3%) for the 2013–2014 season. The corresponding rates for the US population aged 20–64 were 30.5% (4.4%, 49.3%) in 2012–2013 and 7.1%(−5.1%, 32.5%) in 2013–2014. The attack rates in women and men were similar each season. Our findings demonstrate that participatory syndromic surveillance data can be used to gauge influenza attack rates during future influenza seasons.

Influenza causes a substantial burden of illness and severe outcomes in the United States (U.S.) every year. Despite the high burden there is limited information on the attack rates (cumulative incidence of influenza virus infections) for seasonal influenza in the different population groups, as well as limited means for assessing the magnitude of an evolving influenza season. Such information could be useful for informing potential preventative strategies, such as emphasizing the importance of vaccination or the need to seek medical care in certain population groups that feature high contact rates and/or high susceptibility. Current influenza surveillance streams in the United States rely on a variety of data sources including information on outpatient visits to health care providers for influenza-like illness, influenza-associated death reports, laboratory confirmed influenza-associated hospitalizations in children and adults and virological surveillance (number/percent of positive samples by influenza type and subtype) from a variety of public health laboratories^1^. These data sources, although useful, do not allow for the assessment of influenza attack rates in different population subgroups, and their timeliness is not ideal for effective allocation of interventions and resources.

Participatory, community-based syndromic surveillance systems have been introduced across Europe^2^, in Australia^3^ and in the United States^4^ to, amongst many goals, potentially address the limitations of existing healthcare based surveillance in a complementary way. These systems request weekly self-reporting of symptoms from participants, allowing for a longitudinal view of illness burden. The data include a defined denominator (individuals have the option to report if they had none of the symptoms in the past week) and associated demographics which is an advantage compared to traditional influenza monitoring systems, as well as newer data sources from social media or search queries^5^,^6^,^7^. Further, these systems offer a low-cost, convenient surveillance method. Additional data from these systems can potentially be used to address epidemiological questions such as vaccine efficacy^8^, risk factors for influenza-like-illness^9^ and near real-time incidence^10^ which is currently difficult to gauge otherwise, at least in the U.S.

In this paper, we investigate how participatory data can be used to estimate disease burden, specifically using Flu Near You (FNY) surveillance data to estimate U.S. influenza attack rates. Flu Near You is an online participatory syndromic surveillance tool in the United States and Canada^4^ whose participants are sent a short weekly survey via email or a smartphone push notification asking if they experienced any of the 10 select symptoms (fever, cough, sore throat, shortness of breath, chills/night sweats, fatigue, nausea or vomiting, diarrhea, body aches and headache). Participants can also choose to report weekly for their household members. Flu Near You also asks users whether or not they have been vaccinated for the current influenza season each week (they can respond “Yes”, “No”, “Unknown” or not reply), until the answer is affirmative.

Flu Near You has run for three seasons (2011–2012, 2012–2013 and 2013–2014) with sufficient data collected during the last two seasons to estimate influenza attack rates in certain population cohorts defined by age and gender. Briefly, the inference method in Ref. 10 requires the assessment of baseline rates of influenza-like illness (ILI, defined as presence of self-reported fever and cough or sore throat) during periods of low influenza circulation. Excess ILI (rates above the baseline) during periods of active influenza circulation are then converted to influenza attack rates (the cumulative incidence of influenza virus infections) via estimates of the probability *P(ILI|Flu)* of self-reported ILI for influenza cases. This probability is assessed using separate data from the Hong Kong household studies^10^,^11^.

## Results

As of April 1, 2014 there were 143847 total users (including household members) registered in Flu Near You. Of these, there were 1129 participants aged 20–64 included in the 2012–2013 cohort defined by conditions A)–C) and 3189 in 2013–2014. The cohort was 33.7% male and 55.9% female in 2012–2013, and 37.7% male and 46.7% female in 2013–2014. Other demographics and information about the participants and these cohorts is reported in Supplementary Tables S1, S2 and S3.

**Figure 1:**
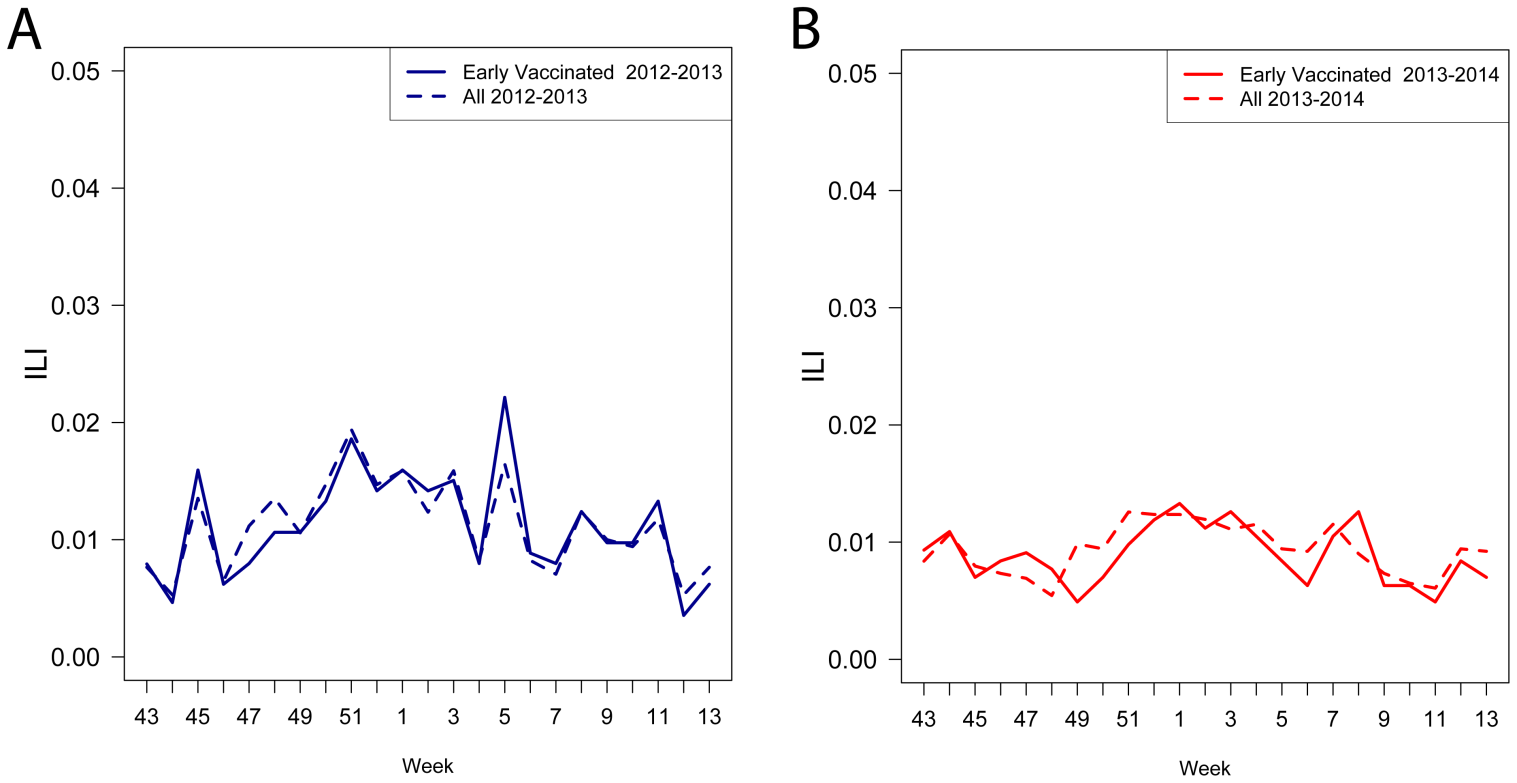
ILI reports for the age 20–64 cohort. Individuals selected were registered by week 43 and reporting at least half of the surveys between week 43 and 13 for everyone (dashed lines), and those that were vaccinated by week 45 (solid lines) (a) 2012–2013 season, (b) 2013–2014 season.

Weekly ILI incidence for the age and gender cohorts is illustrated in Figure 1 and Supplementary Figure S1. Overall, the 2012–2013 season had a higher ILI incidence as illustrated in Figure 1, which is similar to the U.S. CDC data for those seasons^12^,^13^. This trend was similar for both men and women individually (Supplementary Figure S1).

Table 1 gives estimates of the influenza attack rates in the different FNY cohorts (defined by age and gender) of the early vaccinated individuals during the 2012–2013 and 2013–2014 seasons. Additionally, Table 1 presents estimates of influenza attack rates for the corresponding groups (defined by age and gender) in the US population. We also note that that ILI incidence during the baseline period was consistent between seasons (Figure 1 and Supplementary Figure S1). Sensitivity analysis with respect to the choice of the baseline period in Eq. 1 is presented in the supplementary information (Supplementary Table S4).

**Figure 2:**
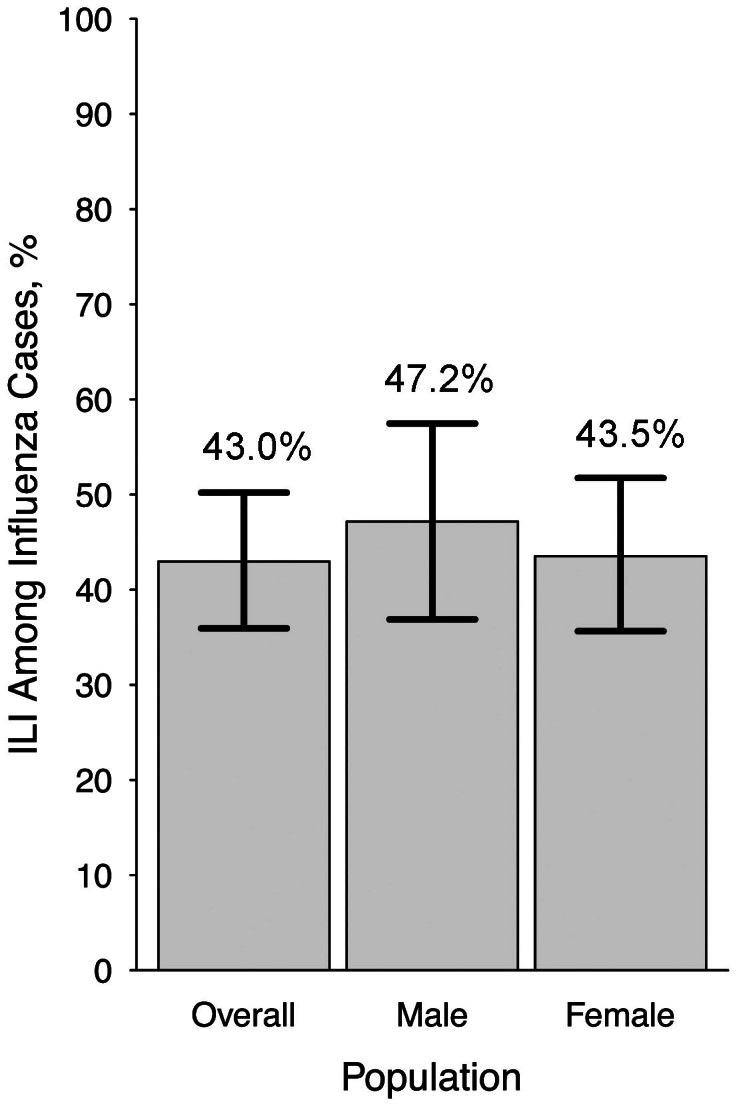
Estimated probability of self-reported ILI for influenza cases in 20–64 age category overall and for men and women separately. The probability is estimated using data from PCR-positive individuals in Hong Kong household studies as described in Ref. 10.

Figure 2 shows the probability of self-reported ILI for influenza cases in adults aged 20–64, and for those by each gender separately. Using data from PCR-positive individuals in Hong Kong household studies as described in Ref. 10, these results shows that estimates of *P(ILI|Flu)* were very similar for women and men.

## Discussion

Participatory surveillance systems, such as Flu Near you in the US and Influenzanet in Europe, can be used to complement the more traditional disease surveillance streams to enhance our understanding of disease dynamics. In particular, they allow one to track disease incidence in defined population cohorts, which is different from e.g. the US CDC surveillance data where only individuals seeking medical attention are accounted for, or Google Flu Trends, where non-specific information from individuals who perform online searches is available. Here, we demonstrate the utility of participatory surveillance systems by adopting the method from Ref. 10 to estimate influenza attack rates during the 2012–2013 and the 2013–2014 seasons for the 20–64 age group in the US using syndromic surveillance data collected in the Flu Near You platform. In this study, the methodology developed in Ref. 10 is further adjusted by including data on vaccination for Flu Near You participants, as well as estimates of vaccination coverage and vaccine effectiveness in the US population. We have found that attack rates in that age group were higher during the 2012–2013 season than during the 2013–2014 season (which is consistent with the U.S. CDC data for those two seasons), and that males and females had similar influenza attack rates. Usage of the joint baseline rate of ILI during weeks of low influenza circulation for both seasons further supports the possibility of utilizing Flu Near You data for the estimation of influenza attack rates during future seasons through the inference framework we have presented, as well as in real-time.

While we restricted the estimation of influenza attack rates to the 20–64 age group, future estimates can be performed for other age groups as well, provided that the corresponding cohorts in Flu Near You would be sufficiently large, and that one obtains reliable estimates of the probability *P(ILI|Flu)* of self-reported ILI for influenza cases in those age groups. Our current estimation of *P(ILI|Flu)* is based on data collected through household studies in Hong Kong^11^, with very little information on symptoms of influenza cases among the elderly available in those studies. We also note that we are not aware of other analogous studies that could provide one with the needed estimates of *P(ILI|Flu)*. Moreover we hope that the thoroughness of the study design in Hong Kong (e.g. the fact that 3 RT-PCR tests were administered for all household contacts, decreasing the bias that might stem from the correlation between the likelihood of influenza virus detection and symptom presentation) should contribute to the reliability of those estimates, at least in that setting. At the same time, it is unclear whether the probability of reporting ILI is the same for Flu Near You participants versus Hong Kong household members – that issue might be particularly problematic for the collection of reports on symptoms in children. Future, context-specific efforts on collection of data on symptoms for individuals in different age groups infected with different influenza sub-types in a manner that is compatible with symptom reporting in online syndromic surveillance platforms like Flu Near You should help address those issues.

The inference method used here has a number of limitations. One, already mentioned in the previous paragraph, is the uncertainty about the estimation of the probability *P(ILI|Flu)* of self-reported ILI for influenza cases. Another is the ambiguity of the key assumption that we make, that rates of ILI not associated with influenza are constant throughout the season. While we have no data to assess this, we were able to address this in a previous study^10^, where ILI rates reported under minimal influenza circulation during a belated influenza season in the Netherlands were temporally constant. We hope to confirm this using Flu Near You data for future seasons. Given the small number of unvaccinated individuals in Flu Near You, we had to perform the estimation of the attack rates via the cohort of vaccinated individuals in FNY, with the extrapolation to the US population relying on the US CDC estimates of coverage rates and vaccine effectiveness. Vaccine effectiveness estimates might be the most tenuous aspect of that extrapolation as the CDC estimates are based on observational data and refers to effectiveness against symptomatic, physician-attended disease while we are interested in effectiveness against influenza infection. Yet another limitation related to the above extrapolation is that online participatory systems may be constrained in terms of the demographics of the participating populations^4^. While women are somewhat overrepresented among Flu Near You participants, we have found that estimates of *P(ILI|Flu)* were very similar for women and men, and that influenza attack rates estimates were also similar, so that overrepresentation of women should not bias the estimation of the influenza attack rates in the overall population. There are other potential sources of heterogeneity, e.g. ones having to do with the geographic distribution of participants and their underlying health conditions. We hope that the continuing growth of the pool Flu Near You participants (exemplified by the 2.8-fold increase in the cohort size for the 2013–2014 season compared to the 2012–2013 season) would help ameliorate the potential biases stemming from that heterogeneity. Finally, we present the estimation of the influenza attack rate for calendar weeks 47–13 of each season, in part due to a potential decrease in the willingness to file reports during the later weeks among the Flu Near You participants. While in 2012–2013 influenza season activity had largely waned by week 12 of 2013^12^ and the above estimates can essentially be interpreted as the whole-season attack rates, there was a later, albeit significantly smaller wave of influenza activity largely driven by influenza B during the 2013–2014 season^13^ and the attack rate during that wave is largely unaccounted for in our paper.

Overall we believe that, notwithstanding certain limitations, data gathered through the Flu Near You participatory surveillance system would allow for estimation of influenza attack rates in different population cohorts during future influenza seasons, rendering a viable surveillance stream for influenza activity in the U.S. This estimation could be performed in real time, provided that vaccine effectiveness estimates are made available, or that a sufficiently large cohort of unvaccinated Flu Near You participants is recruited. Moreover we hope that future growth of the pool of Flu Near You participants and additional efforts on ascertainment of symptoms of influenza cases would help sharpen estimates of influenza attack rates.

## Methods

### Flu Near You Cohorts

We use data on self-reported symptoms among participants in the Flu Near You (FNY) surveillance platform (flunearyou.org) in the United States during the 2012–2013 and 2013–2014 influenza seasons. For each season, the early-vaccinated cohorts in our analyses were defined as individuals aged 20–64 (age-only analysis) or males only or females only (ages 20–64) who have:

- Filled out at least one report by calendar week 43.
- Subsequently filled out reports for at least half of the weeks between calendar week 43 and week 13 of the following year.
- Reported that they did get an influenza vaccination by calendar week 45 of the season.

We restricted our analysis to the early vaccinated individuals in the 20–64 age group because of the small size of other age cohorts, and the small number of unvaccinated FNY participants (Supplementary Tables S1 and S2). To estimate incidence rates in the general population we conducted an extrapolation analysis for which we used corresponding available vaccine efficacy and coverage data from the CDC.

### ILI incidence rates

For each individual filling out a report on a given week *t*, we defined presence of self-reported ILI for that individual to be presence of fever and either cough or sore throat in the weekly report. Because an ILI episode may overlap two consecutive calendar weeks, to avoid double-counting of a single ILI episode we have discarded weekly ILI reports for individuals who have also reported ILI during the preceding week (in the few cases where individuals reported ILI for three consecutive weeks, we did not remove the third week, only the second, from the data). Given that the likelihood of an independent ILI report for a second consecutive week is quite low, the above procedure largely amounts to removal of double reporting (see Ref. 10). We define the ILI incidence rate *ILI*(*t*) in a cohort on a given week *t* to be the number of individuals in the cohort reporting ILI for that week (with double-reporting removed, as explained above) divided by the cohort size^10^.

### Baseline ILI rates

We defined the baseline period each season to be calendar weeks 43–46 due to limited influenza circulation during that period as suggested by the U.S. CDC data^12^,^13^. The period of active influenza circulation we have considered were weeks 47 through week 12 of the following year. Figure 1 suggests that the baseline ILI rates during weeks 43–46 were similar for the 2012–2013 and the 2013–2014 seasons. We therefore define the cohort-specific (e.g. women 20–64) baseline ILI rate *Base* for both seasons as:
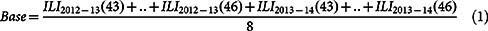
Sensitivity of the attack rate calculation based on using other baseline periods was evaluated (see Supplementary Information).

### Inference of influenza attack rates in FNY

Besides data on weekly ILI rates and the assessment of the baseline rate, inference of a influenza attack rate in a cohort requires an estimate of the probability *P(ILI|Flu)* of self-reported ILI given influenza infection. The latter probability is estimated using data from PCR-positive individuals in Hong Kong household studies as described in Ref. 10, in which self-reported symptom profiles and laboratory confirmed influenza status was measured for a group of individuals whose ages and genders were also recorded. Figure 2 exhibits *P(ILI|Flu)* estimates for all individuals aged 20–64, as well as for females and males in that age category.

The influenza attack rate *AR_flu_* for a given season between calendar week *t* = 47 and week *N* of the following year is estimated as:
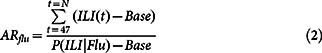
Briefly, the numerator in eq. (2) is the excess ILI rate (above the baseline) during the period of active influenza circulation. The denominator is the excess probability of reporting ILI for influenza cases compared to non-influenza cases (*P*(*ILI*|*Flu*) − *Base*), from which the influenza attack rate is estimated (with more details provided in Ref. 10). As in Ref. 10, posterior samples for each of the quantities in eq. 2 (e.g. *ILI*(*t*)) are independently extracted to get a posterior sample of estimates for *AR_flu_*, for which the mean and the 95% credible intervals are reported.

### Extrapolation to U.S. population

In order to extrapolate the estimation of influenza attack rates from the early vaccinated group of Flu Near You participants to the US population, we split the US population aged 20–64 into three groups: *G_1_* (those who got vaccinated by mid-November), *G_2_* (those who got vaccinated later), and G_3_ (those who never got vaccinated). Since the CDC data on vaccine coverage and effectiveness suggests differences among 18–49 and 50–64 year olds, we further split *G_1_* into *G_11_* (20–49 year olds in *G_1_*) and *G_12_* (50–64 year olds in *G_1_*); similarly, we split *G_2_* into *G_21_* and *G_22_*, and *G_3_* into *G_31_* and *G_32_*. We estimate the size *p_ij_* of each of those 6 groups (as a proportion of 20–64 year old US population), and the attack rate *AR_ij_* in each group. The attack rate among 20–64 year olds is then estimated as the weighted average of those attack rates. Full details are reported in the Supplementary Text S1 section.

Statistical analyses were performed using R version 2.15.1 (R Development Core Team, Vienna, Austria).

## Supplementary Material

Supplementary Information Supplementary Information

## Figures and Tables

**Table 1:** Attack rates for the Flu Near You early vaccinated participants and attack rate estimates extrapolated for the entire U.S. population during the 2012–2013 and 2013–2014 seasons

Cohort	Season, Calendar Weeks	Estimated Attack Rate % (95% CI)
Early Vaccinated 20–64 (FNY)	2012–2013, 47–12	14.7 (5.9, 24.1)
Early Vaccinated 20–64, men (FNY)	2012–2013, 47–12	18.8 (3.5, 34.9)
Early Vaccinated 20–64, women (FNY)	2012–2013, 47–12	20.2 (8.7, 32.9)
Early Vaccinated 20–64 (FNY)	2013–2014, 47–12	3.6 (−3.3, 10.3)
Early Vaccinated 20–64, men (FNY)	2013–2014, 47–12	4.8 (−7.1, 15.8)
Early Vaccinated 20–64, women (FNY)	2013–2014, 47–12	4.7 (−3.8, 13.1)
Overall 20–64 (US)	2012–2013, 47–12	30.5 (4.4,49.3)
Overall 20–64 (US)	2013–2014, 47–12	7.1 (−5.1,32.5)
Men 20–64 (US)	2012–2013, 47–12	36.9 (3.0, 80.9)
Men 20–64 (US)	2013–2014, 47–12	10.1 (−17.8,39.5)
Women 20–64 (US)	2012–2013, 47–12	37.9 (7.4,75.6)
Women 20–64 (US)	2013–2014, 47–12	9.6 (−9.5,32.75)
