# Supplementary material for: Estimating influenza attack rates in the United States using a participatory cohort
Source: Sci Rep. 2015 Apr 2;5:9540. doi: 10.1038/srep09540 (PMC4894435; doi:10.1038/srep09540)
Supplement: Supplementary Information [file srep09540-s1.doc]

Supplementary Information

Manuscript Title: Estimating influenza attack rates in the United States using a participatory cohort

Author List: Rumi Chunara, Edward Goldstein, Oscar Patterson-Lomba, John S. Brownstein

**Supplementary Text S1. Details of attack rate extrapolation to US population.**

In order to extrapolate attack rates in the general population for vaccinated cohorts, we have combined our estimate of the attack rate among vaccinated individuals with the US CDC data on vaccination coverage (both early and full-season ones) and vaccine effectiveness to extrapolate attack rates in the general populations. Details of this method are below:

We split the population aged 20-64 into three groups

*G­_1_* – those who got vaccinated early (by mid-November)

*G_2_* – those who got vaccinated later

*G_3_* – those who never got vaccinated

Since the CDC data on vaccine coverage and effectiveness suggests differences among 18-49 and 50-64 year olds, we further split *G_1_* into *G_11_* (20-49 year olds in *G_1_*) and *G_12_* (50-64 year olds in *G_1_*); similarly, we split *G_2_* into *G_21_* and *G_22_*, and *G_3_* into G_31_ and *G_32_*. We estimate the size $p_{ij}$of each of those 6 groups (as a proportion of 20-64 year old US population, thus ), and the attack rate $AR_{ij}$ in each group. The attack rate among 20-64 year olds is then estimated as the weighted average of those attack rates

(1)

More precisely, we proceed as follows:

1. To estimate, say $p_{21}$during the 2012-2013 season, we use data from <http://wonder.cdc.gov/Bridged-Race-v2013.HTML> to conclude the 20-49 year olds constituted a proportion x=0.675 of the 20-64 year old US population in 2012 (for male-specific estimates we would use x as the proportion of 20-49 year old men in the 20-64 year old male US population). We subtract the early-season vaccination coverage estimate for 18-49 year olds in <http://www.cdc.gov/flu/fluvaxview/nifs-estimates-nov2013.htm>, Table 3, from the end-season estimate in <http://www.cdc.gov/flu/fluvaxview/coverage-1213estimates.htm>, Table 3, to posit a coverage rate of y=0.311-0.263=0.048 between mid November to the end of the season among 20-49 year olds. Thus we estimate

$p_{21}=x\cdot y=0.0324$.

We note that there are no end season estimates for the 2013-2014 vaccination coverage yet. To estimate$p_{21}$ during the 2013-2014 season, we take up the 2012-2013 estimate for the late season vaccination rate y=0.048 among 20-49 year olds for the 2012-2013 season as well. To estimate $p_{32}$ during the 2013-2014 season, we add the early season coverage estimate (0.314) to the late season coverage (of 0.048) to estimate the coverage proportion of 0.364 for 20-49 year olds in 2013-2104. This translates into an estimate of 0.636 for the proportion of unvaccinated, etc.

2. To estimate the attack rates each season, let EAR be the estimated attack rate among the early vaccinated 20-64 year olds in FluNearYou (overall, or gender specific). Let q and 1-q be, respectively, the proportions of 20-49 and 50-64 year olds among the early vaccinated 20-64 year old individuals in the FluNearYou cohort considered (overall or by gender). Let and be, respectively, the vaccine efficacies for 20-49 and 50-64 year olds during a given season (which are found from the adjusted overall estimates for those age groups in <http://www.cdc.gov/mmwr/preview/mmwrhtml/mm6207a2.htm> and <http://www.cdc.gov/mmwr/preview/mmwrhtml/mm6307a1.htm>).

We first estimate $AR_{uv}=AR_{31}=AR_{32}$ among unvaccinated individuals 20-64. Since the attack rates among the early vaccinated individuals aged 20-49 and 50-64 are $AR_{31}(1-VE_{1})$ and $AR_{32}(1-VE_{2})$ correspondingly, we can estimate $AR_{uv}$ from the following

which means

(2)

We then estimate

(3)

While there is no principled way of estimating $AR_{21}$, we simply assume that it is the average

$AR_{21}=\frac{AR_{11}+AR_{31}}{2}=EAR\cdot\frac{1-(VE_{1}/2)}{q(1-VE_{1})+(1-q)(1-VE_{2})}$ (4)

with analogous estimates for $AR_{12}$ and $AR_{22}$. We note that all estimates in eqs. 2-4 are multiples of EAR. Then we plug them into eq. (1) to get that

$AR=EAR\cdot D$ (5)

where D is a number estimated from several CDC data sources (population, vaccination coverage, vaccine effectiveness).

| Season | Total cohort* registered by week 43 and filled in half surveys between 43 and 13 | Number (Percent) of < 20 years old participants | Number (Percent) of 20-64 participants | Number (Percent) of 65 years old and older participants | Number (Percent) of Female/Male participants overall |
| --- | --- | --- | --- | --- | --- |
| 2012-2013 | 2467 | 290 (11.7%) | 1700 (68.9%) | 358 (14.5%) | 1455/987 (58.9%/40.0%) |
| 2013-2014 | 7503 | 961 (12.8%) | 4773  (63.6%) | 1386 (18.5%) | 3872/3032 (51.6%/40.4%) |

* Total cohort larger than sum of each group due to invalid dates of birth or no gender information supplied

Supplementary Table S1. Demographics of selected cohort (registered by week 43, and filled in at least half of the surveys between and including weeks 43 and 13.

| Season | Number of 20-64 participants from Table S1 who reported Vaccination “Yes” by week 45 (Percent of aged 20-64 participants) | Number (Percent) of Female/Male participants of those aged 20-64 from Table S1 |
| --- | --- | --- |
| 2012-2013 | 1129 (66.4%) | 631, 381 (55.9%, 33.7%) |
| 2013-2014 | 3189 (66.8%) | 1489, 1203 (46.7%, 37.7%) |

Supplementary Table S2. Those from the aged 20-64 selected cohort (registered by week 43, and filled in at least half of the surveys between and including weeks 43 and 13) who also reported Vaccination “Yes” by week 45 overall, and by gender.

| Season | Reported Vaccination At Any Time “Yes” | Final Report of Vaccination Status “No” |
| --- | --- | --- |
| 2012-2013 | 1946, 1283 | 219, 110 |
| 2013-2014 | 5918, 3578 | 795, 377 |

Supplementary Table S3. End of season vaccination cohort sizes for those with vaccination information available (all ages, ages 20-64)

| Season | Baseline Start (Week) | Baseline End (Week) | Attack Rate (95% CI) |
| --- | --- | --- | --- |
| 2012-2013 | 43 | 46 | 14.7 (5.9, 24.1) |
| 2012-2013 | 42 | 46 | 14.0 (6.9, 23.7) |
| 2012-2013 | 42 | 45 | 12.7 (4.1, 22.0) |
| 2012-2013 | 43 | 45 | 11.8 (2.5, 21.5) |
| 2013-2014 | 43 | 46 | 3.6 (-3.3, 10.3) |
| 2013-2014 | 42 | 46 | 3.8 (-1.9, 9.6) |
| 2013-2014 | 42 | 45 | 2.3 (-6.1, 8.5) |
| 2013-2014 | 43 | 45 | 1.3 (-6.2, 8.5) |

Supplementary Table S4. Attack rates based on different baseline selections

**
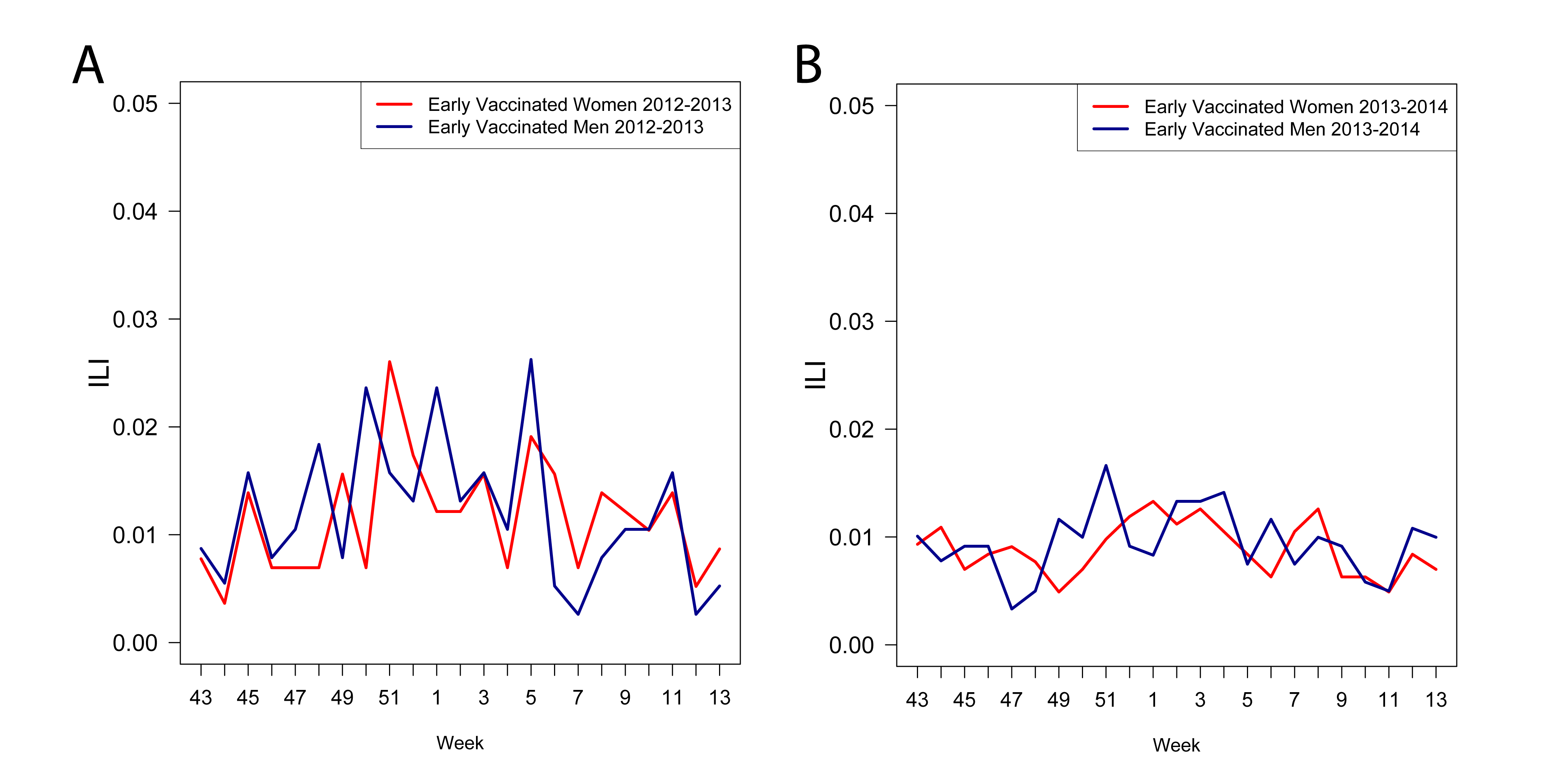
**

**Supplementary Figure S1. ILI reports in the cohort ages 20-64 registered by week 43 and reporting at least half of the surveys between week 43 and 13, by gender.** (A) Reports for women. (B) Reports for men.

**
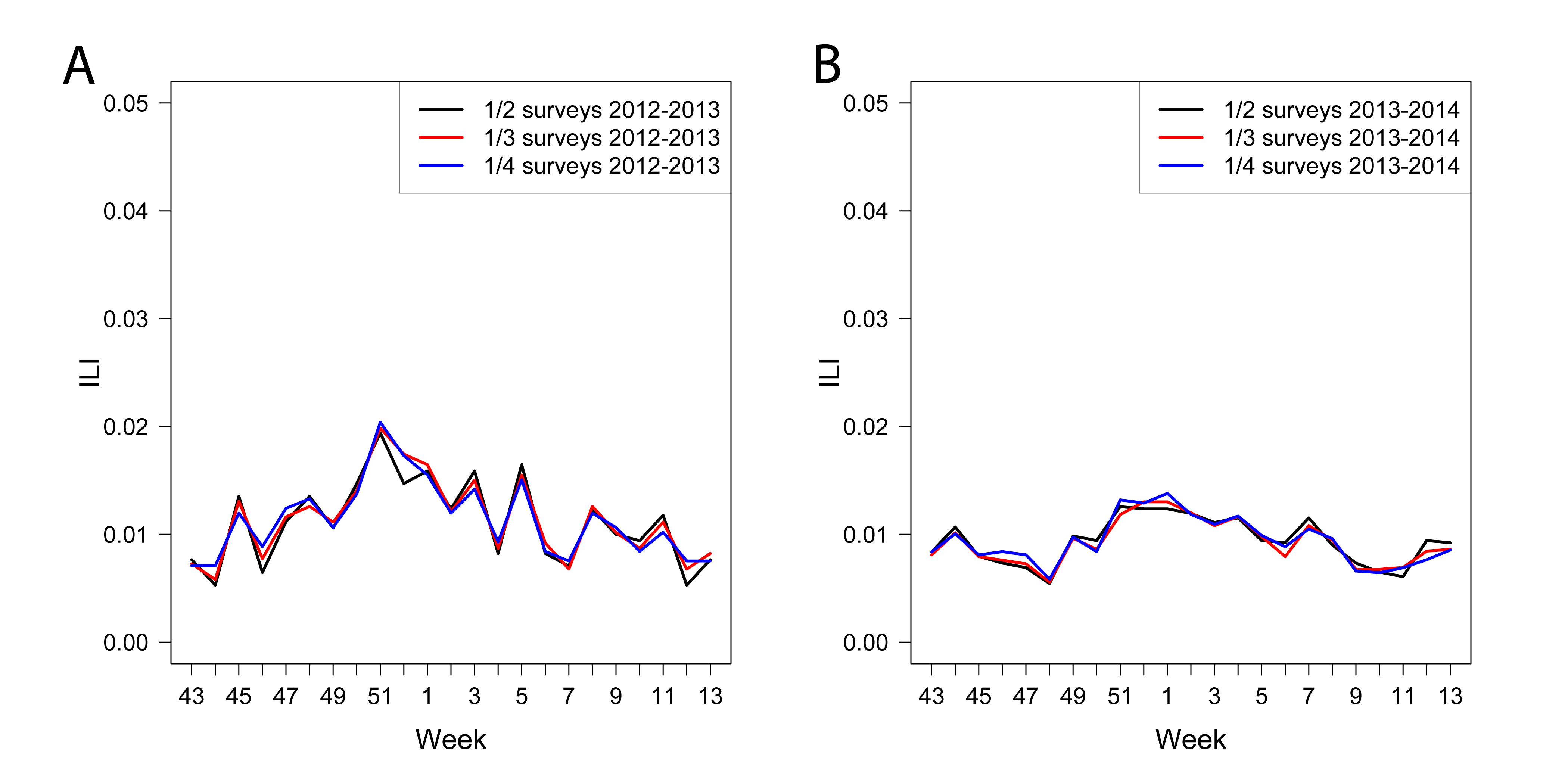
**

**Supplementary Figure S2. ILI reports by cohort registered by week 43 and reporting at least one quarter, third and half of the surveys between week 43 and 13.** (A) Reports from 2012-2013 cohort. (B) Reports from 2013-2014 cohort.
